# Annotations

**Published:** 1889-02-02

**Authors:** 


### ANNOTATIONS.

**The Universal Cure at last**

That there is a "universal cure" if it could only be found seems to be not for a moment doubted by the majority of mankind. The proof of this is seen constantly in the eagerness with which the quack medicines of the day are bought up when they are sufficiently advertised. Any vendor of a pill or an "elixir" who is possessed of sufficient skill and boldness in the art of telling lies, is practically certain of making a fortune within a very short time. A "well-puffed pill" has always been considered a valuable property, and it is quite as valuable to-day as it was in that period of innocence and simplicity "the early part of the century." According to a dental contemporary, there appeared in 1813 a pamphlet published in German, which gave an account of a "stomach brush," which was to cure everything. The brush could be purchased at the brushmakers at the old Court saddler's shop in Broad Street, in Colln-on the-Spree. In form it resembled a pipe-cleaner. The stalk was made of four twisted wires, covered with thread, silk, or small ribbons, and was 26in. long. The brush was 2in. long, and 1½in. broad, and was made of hair from a goat's beard. Those trained in the use of the goat's beard article soon learnt to endure one made of horsehair. The brush was to be used every morning, and its use' followed by sufficient libations of cold brandy and water. Whoever used it was entirely independent of any other remedy. It was "good against cold, hot or poisonous fevers." It gave "a good appetite for eating;" cured "asthma, hemorrhage, headache, chest complaints, coughs, consumption, apoplexy, toothache, sore eyes, dysentery, quinsey, ulcers, abcess, cardialgy." It "favoured digestion, strengthened the heart, drove away pimples in the skin, cured choking in the stomach, made fat people thin, and thin people fat," and generally did anything and everything which a well-conducted and thoroughly reliable "universal medicine" ought to do. Nothing is said of a list of cases, nor are there any testimonials now available. But there need be no doubt that if any brushmaker will bring a proper "stomach-brush" into the market, and get it properly "puffed," he will soon be able to record as many cures and to publish as many testimonials as he pleases. Is it of any use to point a moral? Probably not. The public has always displayed a warm admiration for cheats. There must be a certain satisfaction in being imposed upon; otherwise imposition would not continue to be so profitable a trade. Or is it that people think they have a right to be poisoned if they please, and that they are willing to risk the poisoning in order to demonstrate the independence of their minds.

**"Physicians' Whims," or Women's Faults?**

Last week the *Standard* did us the honour of writing a critical commentary on a little homily which appeared in The Hospital under the title "Coaxing the Appetite." Putting himself in the place of his readers, the writer of the article asked what patients are to do among so many diverse medical opinions? Five-and-twenty years ago, "starvation" was in vogue; to-day, "rational and sufficient feeding" carries the votes of the leading physicians. It is probable, says the *Standard*, that "stinting" will again have its turn, and it therefore concludes, with philosophic stoicism, that "physicians' whims" must be endured. Not at all. It may be pointed out that, so far as The Hospital was concerned, the class of people called "patients" were not under discussion. It was not the sick in charge of physicians upon whom the duty of coaxing the appetite was urged. The real subjects of the medical preacher's exhortations were those who suppose themselves, and are supposed by their friends, to be well, but who, in consequence of the indulgence of a feeble and unworthy "table habit," have sunk into circumstances of chronic weakness of body, irregularity of temper, and mental inconsequence. It is possible that some few persons of this class may be the victims of "physicians' whims," for even physicians are not always wise, and there have been known those in high places whose "whims" have been more conspicuous than their wisdom. But the last quarter of a century has to a large extent certainly "changed all that." Most of the offenders to whom our friendly warnings were addressed have no one but themselves to blame. There is a class of persons, largely composed of women, who have money without work or responsibility, and who constantly live on the border-lands of ill-health. If they are not "ill," as certainly they are not "well." They are not happy in themselves, they are not pleasant to others, and they are decidedly not of any particular use in the world. Other people have to endure them. Now, these people do not deserve pity. Sharp and rousing censure is what they merit and require. They are doing none of the good and useful work they might be doing if they would acquire the physical and mental energy which are within their reach, and they are, moreover, inflicting a great injury upon those relatives, friends, and servants who have to bear with their incapacity and querulousness, and to be the poorer by the lack of their service. Many a wife and many a daughter of this class has just turned the scale to the side of family adversity, instead of prosperity, by the very habits we complain of. The creatures themselves do not suffer much, because their brains become so weakened and their sloth so pronounced that they lose their capacity for suffering. It is the toiling husband and the disappointed father upon whom the consequences fall. Physicians' prescriptions are of no use to such people. The disease is a moral fault—a failure of duty; and until that is cured the doctor will prescribe in vain. It would be a great gain to the community if physicians themselves would recognise this undoubted and widespread evil, and treat it with that simplicity and thoroughness which characterise the best medical practice in other departments.

**What is "Pap"?**

Commenting on an article which appeared in this journal a week or two ago on the "papoid" nature of much of the food of the present day, the *Christian World* very pertinently asks, "What is pap?" If the querist were a Yorkshireman or an American, or had been a diligent student of *Punch* during the last quarter of a century, he would have been able to answer the question himself as well as to ask it. Englishmen happily have a fine fund of common sense, and, provided the meaning of a term be made clear to them they do not, like the French, worry themselves about a rigid definition. We will not, therefore, attempt an academic equivalent of "pap," but rather endeavour to furnish a brief general exposition which may be of practical service. "Pap" among the common people in the North Riding of Yorkshire is mother's milk. When the baby of a farm labourer's wife cries, the good woman generally concludes that it wants its "pap," and accordingly she gives it the breast. From this beginning the use of the word has extended itself until it has taken in all kinds of "baby's food," and finally every variety of food used by adults which can claim similarity or kinship with it. The various prepared foods so commonly supplied to "infants and invalids," the beef teas, mutton broths, calfs' foot jellies, egg-flips, and other necessaries of the sick-room, are all more or less of the nature of "pap." That is, they are generally so made as to be easy and quick of digestion. They are, therefore, in every sense of the word, usually the very best things which can be given to "infants and invalids." But, according to the American contemporary from which we originally quoted, the use of these "papoid" foods is rapidly extending among those who are neither "infants nor invalids." Therein American physicians see a distinct rock ahead. It is well known that chronic dyspepsia is as much a national characteristic of Americans as a "nasal twang" or a desire to "lick creation." How much of this is due to the papoid habit? Looking homewards, it seems certain that in this country we are rapidly giving up the substantial, if coarse, fare of our grandfathers. The coarseness may be dispensed with, but not the substantiality. For ordinary persons two or three substantial meals a-day, and time to digest them, are absolutely necessary. Not only are they necessary for outdoor workers, but probably still more for those who work indoors and with their brains. The present writer is distinctly of opinion, both from personal and professional experience, that what has been called "papoid" food tends ultimately to diminish the energy of the brain, to promote "nervousness," and especially to produce that most distressing form of stomach worry, nervous dyspepsia. Those who take "papoid" food thereby gain the dangerous facility of returning to mental work immediately after a full meal. That facility is purchased at a cost which is incalculable. For a person in average health nature demands two or three substantial meals daily, *and time to digest them.* If this rule be persistently departed from, it can only be in most cases at the ultimate cost of ruined digestion, enfeebled brains, and premature old age.

